# Genome-wide identification of *TBL* gene family and functional analysis of *GhTBL84* under cold stress in cotton

**DOI:** 10.3389/fpls.2024.1431835

**Published:** 2024-06-18

**Authors:** Xiaoqing Zhu, Xiaowei Ma, Wanying Hu, Yulin Xing, Shengcai Huang, Zequan Chen, Lei Fang

**Affiliations:** ^1^ Zhejiang Provincial Key Laboratory of Crop Genetic Resources, Institute of Crop Science, Plant Precision Breeding Academy, College of Agriculture and Biotechnology, Zhejiang University, Hangzhou, China; ^2^ Hainan Institute of Zhejiang University, Sanya, China

**Keywords:** cotton, *TBL* gene family, fiber quality, *GhTBL84*, VIGS, cold stress

## Abstract

Cotton fiber, the mainstay of the world’s textile industry, is formed by the differentiation of epidermal cells on the outer peridium of the ovule. The *TBL* gene family is involved in the regulation of epidermal hair development as well as response to abiotic stress. However, the function of *TBL* genes in cotton has not been systematically studied yet. Here, we identified 131 and 130 *TBL* genes in TM-1 (*Gossypium hirsutum*) and Hai7124 (*Gossypium barbadense*), respectively. Phylogenetic, gene structure, expression pattern and *cis*-element of promoter analysis were performed and compared. Single gene association analysis indicated that more *TBL* genes related to fiber quality traits were found in *G. barbadense*, whereas more genes associated with yield traits were found in *G. hirsutum*. One gene, *GhTBL84* (GH_D04G0930), was induced by treatment at 4°C for 12 and 24 h in *G. hirsutum* and silencing of the *GhTBL84* gene by VIGS technology in TM-1 can significantly improve the resistance of cotton seedlings to low temperature stress. In sum, our study conducted a genome-wide identification and comparative analysis of *TBL* family genes in *G. hirsutum* and *G. barbadense* and demonstrated a group of *TBL* genes significantly associated with fiber quality and excavated cold stress responsive gene, such as *GhTBL84*, providing a theoretical basis for further improving cotton agronomic traits.

## 1 Introduction

Cotton is a warm-season crop, originating from tropical and subtropical regions, with an optimal growth temperature range of (28 ± 3°C), which still retains its sensitivity to low temperatures after a long period of domestication and selection by humans (Pham et al., 2018). Cotton fiber is a unicellular hair-like structure formed by a single cell protrusion from the ovule epidermis, which in turn differentiates into a highly elongated and thickened single cell of seed trichomes (Pham et al., 2018; Zhai et al., 2023). The fiber development can be broadly divided into four sequential and overlapping stages, mainly in terms of days post anthesis (DPA): cell initiation (-3 to 3 DPA), cell elongation (3 to 20 DPA), secondary cell wall (SCW) synthesis and thickening (20 to 45 DPA), and dehydration maturation (45 to 50 DPA) (Haigler et al., 2012). The number of fiber cell initiations determines cotton fiber yield, while cell elongation and cell wall thickening determine final fiber quality. *G. hirsutum* bolls are large, high yielding and adaptable, but their fiber quality is poor, while *G. barbadense* bolls are small, low yielding and late maturing, but their fiber quality is excellent and they are a very good raw material for spinning high count yarn (Avci et al., 2013).

The DUF231 (Domin of unknown function) family are proteins that contain a conserved TBL (trichome birefringence like) structural domain that is unique to plants. Studies have shown that TBL proteins can act as polysaccharide O-acetyltransferases and catalyse the O-acetylation of specific cell wall polymers (Yuan et al., 2016a; Stranne et al., 2018, b). *TBL3* and *CESA* genes are transcriptionally coordinated, and knockdown of *TBL3* reduces crystalline secondary wall cellulose in trichomes and stems in *Arabidopsis* (Bischoff et al., 2010). The *Arabidopsis esk1/tbl32/tbl33* triple mutant shows a significant reduction in cellulose and xylan content, disorganization of secondary cell wall structures and severe collapse of xylem cell structures (Yuan et al., 2016a). In addition, The TBL family of proteins is also involved in the regulation of plant responses to abiotic stresses. The *Arabidopsis esk1* mutant increased plant tolerance to cold stress, with a 5.5°C increase in freezing tolerance (Xin et al., 2007). The cotton *GhTBL34* gene was located in the confidence interval of a major quantitative trait locus for *Verticillium wilt* (VW) resistance, and the VIGS test showed that silencing *TBL34* reduced VW resistance in cotton (Zhao et al., 2021).

Cold stress is an environmental factor that has a significant impact on plant growth, productivity and survival, and changes the fluidity of plant cell membranes when the external ambient temperature is altered (Sangwan et al., 2002). This change may be sensed by membrane-localized proteins, possibly including receptor-like protein kinases and histidine kinases (Ding et al., 2019). The receptor-like cytoplasmic kinase cold-responsive protein kinase 1 (CRPK1) transduces cold signals from the plasma membrane to the nucleus via 14–3-3 and C-REPEAT binding factor (CBF) proteins, affecting freezing tolerance in *Arabidopsis* (Liu et al., 2017). Under cold stress, CBFs (also known as dehydration response element-binding proteins or DREB1) transcription factors from the DREB subfamily A-1 of the ERF/APETALA2 transcription factor family are essential for acquired cold tolerance (Guo et al., 2018; Kidokoro et al., 2022).

In this study, we demonstrated a group of *TBL* genes in the cotton genome and performed phylogenetic, gene structure, expression pattern and promoter element analyses. The *TBL* genes related to fiber quality were identified by single gene association analysis in *G. hirsutum* and *G. barbadense*. In addition, the cold stress responsive *TBL* gene was excavated, such as *GhTBL84*, and silencing the *GhTBL84* gene can improve the cold resistance of cotton. It provides a theoretical basis for further improvement of cotton agronomic traits and abiotic stress resistance in cotton.

## 2 Materials and methods

### 2.1 Plant materials and treatments

*G. hirsutum* cv. Texas Marker-1 (TM-1), the genetic standard line for *G. hirsutum* cotton, was used as the plant material in this study. The plants were cultured in a growth chamber with conditions set to 22°C day (16 h)/night (8 h), 65% humidity and 120 μmol m^−2^ s^−1^ of light intensity. For cold treatment, four-leaf cotton seedlings were transferred to a thermostat incubator and grown at 4°C for 72 hours for phenotypic observations.

### 2.2 Identification of *TBL* gene family and phylogenetic analysis in cotton

The *G. hirsutum*, *G. barbadense*, *G. arboreum* and *G. raimondii* genome sequences were downloaded from COTTONOMICS (http://cotton.zju.edu.cn/index.htm) and CottonMD (https://yanglab.hzau.edu.cn/CottonMD.1). All *Arabidopsis* TBL protein sequences were obtained from TAIR database (https://www.arabidopsis.org/) were used as queries for BLASTP (https://blast.ncbi.nlm.nih.gov/Blast.cgi). The obtained protein sequences of TBLs were submitted to the NCBI conserved domain database (https://www.ncbi.nlm.nih.gov/cdd) for further confirmation.

The TBL members of *Arabidopsis* and four *Gossypium* species were aligned by multiple sequence alignment using MUSCLE (Edgar, 2004). MEGA (v.11.0) software was used to construct a phylogenetic tree using the maximum likelihood estimation (ML) method with a bootstrap set to 1000 times. The phylogenetic tree was further embellished using the online software iTOL (https://itol.embl.de/).

### 2.3 Protein properties, gene structure, conserved motif and *cis*-elements of promoter analysis

The molecular weight (MW) and isoelectric point (pI) of TBLs were obtained by ExPASy (https://web.expasy.org/protparam/). The subcellular location was predicted with Cell-PLoc 2.0 (http://www.csbio.sjtu.edu.cn/bioinf/Cell-PLoc-2/). The gene structures and conserved motifs of TBLs were analyzed using TBtools software (v 2.0) (Chen et al., 2023). For *cis*-elements of promoter analysis, upstream 2,000 bp sequences of the start codon was extracted and submitted to PlantCARE database (Lescot et al., 2002).

### 2.4 Chromosomal location, collinearity and protein-protein interaction network analysis

The chromosomal positions of *TBL* genes were obtained via a gff3 annotation file from COTTONOMICS (http://cotton.zju.edu.cn/index.htm) and displayed by MapChart (v.2.2) (Voorrips, 2002). MCScanX software was used to identify gene duplication and syntenic regions, and the results were visualized by TBtools software (v 2.0) (Chen et al., 2023). The protein interaction network prediction of GhTBLs and GbTBLs was analyzed by the STRING online database (https://cn.string-db.org/) (Szklarczyk et al., 2021).

### 2.5 Association analysis of genes with agronomic traits

The genetic variation information and phenotypic data of *G. hirsutum* and *G. barbadense* populations (Fang et al., 2017; Hu et al., 2019; Fang et al., 2021) were utilized to annotate variant loci using ANNOVAR (Wang et al., 2010). Subsequently, haplotype analysis of *TBL* gene family was conducted based on the typing of nonsynonymous mutations, and t-tests were performed on different haplotypes (*P* < 0.05), thereby eliminating *TBL* genes associated with agronomic traits. The results were visualized with R software.

### 2.6 Quantitative real time-PCR

The qRT-PCR reaction was performed following the instructions of HiScript II One Step qRT-PCR SYBR Green Kit (Q221–01, Nanjing Novozymes Bioscience and Tech-nology Co., Ltd., China). The reaction system consisted of 2 μL of cDNA, 0.4 μL of each upstream and downstream primer, 7.2 μL of ddH2O, and 10 μL of 2 × SYBR Premix Ex-TaqTM. The PCR reaction program was set as follows: 95°C for 3 min, followed by 40 cycles of 95°C for 10 s and 60°C for 30 s, and 95°C for 15 s and 60°C for 1 min. Each reaction was repeated three times, and the relative expression was calculated using the 2^-ΔΔCT^ method (Livak and Schmittgen, 2001). The *GhHis3* gene was selected as the internal reference gene (Mao et al., 2023), and all primer sequences were listed in **Supplementary Table S8**.

### 2.7 Virus-induced gene silencing assay

Silencing fragments of the *GhTBL84* gene were selected using the SGN VIGS Tool (https://vigs.solgenomics.net/) and amplified by specific primers (**Supplementary Table S8**) from cDNA of TM-1 and cloned into a pTRV2 vector. The fusion vector *TRV2::GhTBL84*, *TRV2:CLA1* (positive control) and empty vector *TRV2::00* (negative control), *TRV1::00* were transformed into Agrobacterium tumefaciens GV3101. The final construct was mixed with the helper vector (*TRV1::00*) in the ratio of 1:1 with OD600 = 2.0. Finally, the mixture was injected into the cotyledons of cotton TM-1 (Li J. et al., 2023).

## 3 Results

### 3.1 Identification of *TBL* gene family

Amino acid sequence similarity and conserved structural domains were identified in TBLs from four different cotton species: *G. hirsutum* (131), *G. barbadense* (130), *G. arboreum* (66), and *G. raimondii* (62). The predicted protein length and molecular weight (MW) of the cotton *TBL* genes ranged from 221 aa/40.11 kDa to 1,422 aa/99.13 kDa, while the theoretical isoelectric point (pI) ranged from 5.18 to 9.51. Subcellular localization predictions indicated that TBLs are distributed in various tissues of both *G. hirsutum* and *G. barbadense*. GhTBLs are mainly located in chloroplasts, indicating their potential involvement in regulating various cellular functions and playing a crucial role in photoregulation. It is worth noting that GbTBLs are primarily situated in the cell wall, while GbTBLs are believed to be associated with the development of cotton fibers. Detailed information about the predicted protein length, pI, MW, chromosomal location, and other related information is shown in **Supplementary** **Tables S1** and **S2**.

### 3.2 Phylogenetic analysis of *TBL* gene family

**Figure 1:**
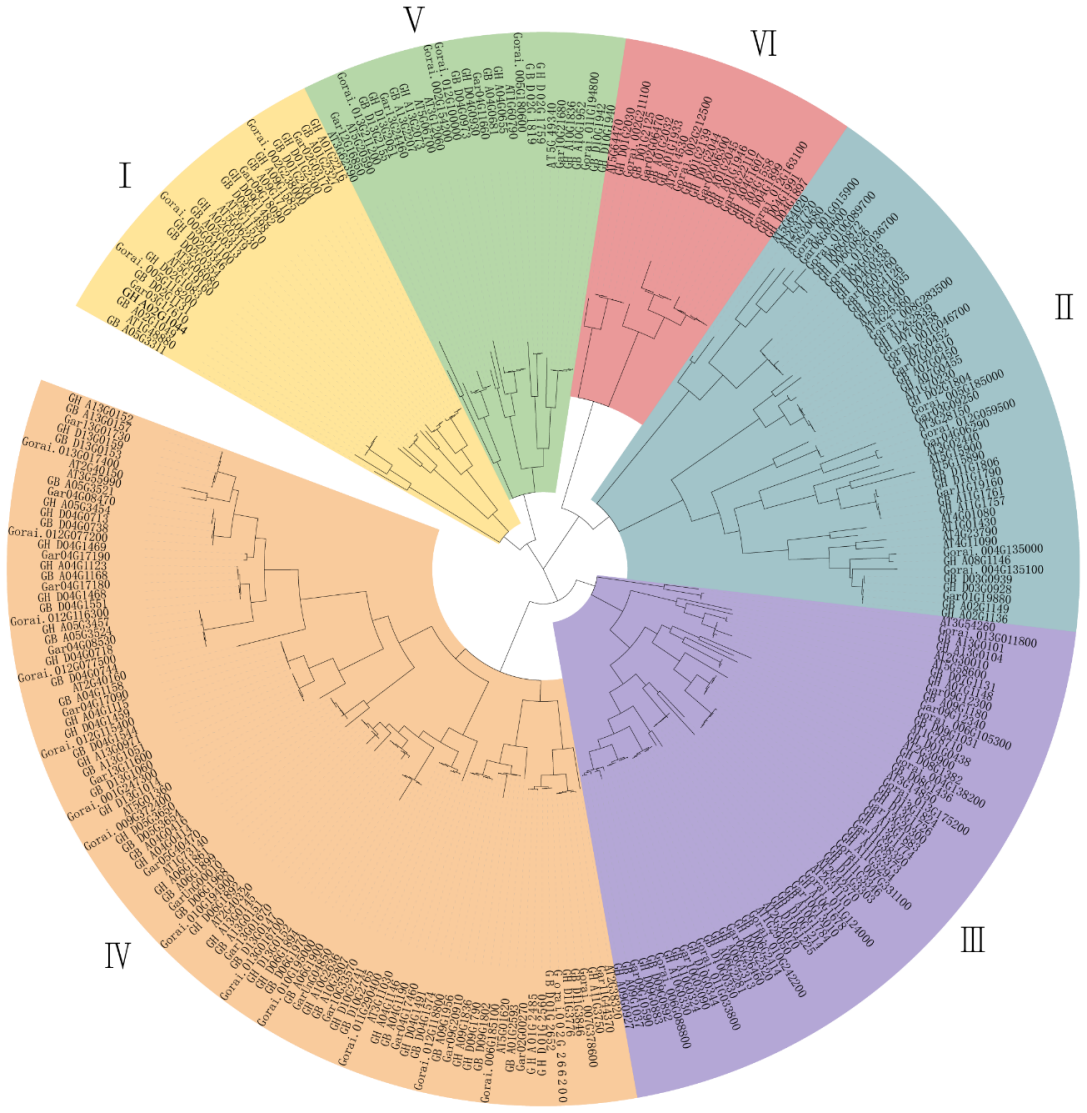
Phylogentic analysis of *TBL* gene family from *Arabidopsis thaliana* (AT), *G. hirsutum* (GH) and *G. barbadense* (GB), *G. arboreum* (Ga), *G. raimondii* (Gr). The phylogenetic tree was constructed using the maximum likelihood estimation (ML) method by MEGA 7.0 software.

To determine the evolutionary relationships of the cotton *TBL* gene family, a phylogenetic evolutionary tree was constructed using the *Arabidopsis thaliana TBL* gene family as a reference (**Figure 1**, **Supplementary** **Table S3**). The *TBL* gene family was classified into six subgroups (Groups I to VI) based on their affinities, which is consistent with previous reports (Stranne et al., 2018). Group IV has the largest of members, with 99 members, including 9 in *A. thaliana*, 30 in *G. hirsutum*, 29 in *G. barbadense*, 15 in *G. arboreum*, and 16 in *G. raimondii*. In contrast, Group VI has the lowest number of members, with only 21 members, including 2 in *A. thaliana*, 7 in *G. hirsutum*, 6 in *G. barbadense*, 3 in *G. arboreum*, and 3 in *G. raimondii*. The number of members in each subgroup were varied. However, each group contained an approximately equal number of *G. hirsutum* and *G. barbadense* members, which appeared in corresponding pairs. In addition, the number of *G. hirsutum* and *G. barbadense* members was nearly twice that of the diploid ancestral species, *G. arboreum*, and *G. raimondii*, consistent with the evolutionary relationship of the species. Five genes from *G. hirsutum* (GH_A01G1358, GH_A11G3750, GH_D04G1469, GH_D04G1899, GH_D09G0883) and four genes from *G. barbadense* (GB_A04G1681, GB_A09G1180, GB_D01G0463 and GB_D09G1031) were identified as lacking corresponding homologous genes, suggesting gene loss in some *TBLs* during the evolution (**Supplementary** **Table S4**).

### 3.3 Conserved motifs and gene structures of *TBL* gene family

Ten motifs were identified in the *TBL* gene family of *G. hirsutum* and *G. barbadense* using the online tool MEME to predict conserved motifs. The conserved motifs of homologous genes in subgroups A and D of *G. hirsutum* and *G. barbadense* are similar in terms of their types, numbers, and distribution locations (**Supplementary Figure S1**). Motifs 4, 5, and 9 are present in both *G. hirsutum* and *G. barbadense*, indicating a high degree of conservation during evolutionary. Motif 1 is present in all family members except for GH_A08G1146. Furthermore, all contained motif 2 except for GH_A05G3406, GH_A06G1862, GB_A08G1294, and GB_D09G1498, suggesting that some of the *TBL* genes have lost motifs during evolution, which may have led to functional changes. Analysis of the exon and intron structures of the *TBL* genes showed that the genes within the same group had similar gene structures, with comparable exon lengths and distributions, indicating the reliability of the phylogenetic classification. However, individual genes, GH_D11G1790/GB_D11G1806 and GH_A11G1271/GB_A11G1277, have different gene lengths but encode amino acid sequences of the same length (**Supplementary** **Table S4**), suggesting that introns have undergone selective splicing during evolution, leading to gene functional diversity.

### 3.4 Chromosomal location and gene duplication of *TBL* gene family

**Figure 2:**
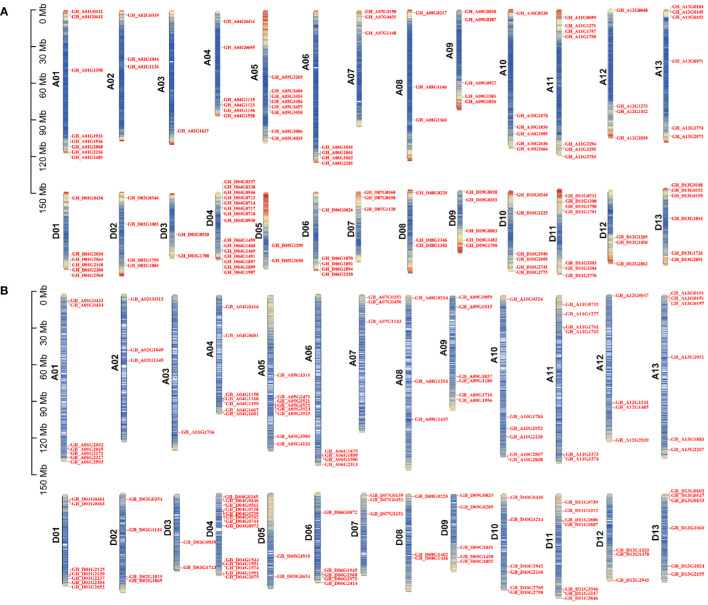
Chromosome location analysis of *TBL* gene family of *G. hirsutum* **(A)** and *G. barbadense* **(B)**.

**Figure 3:**
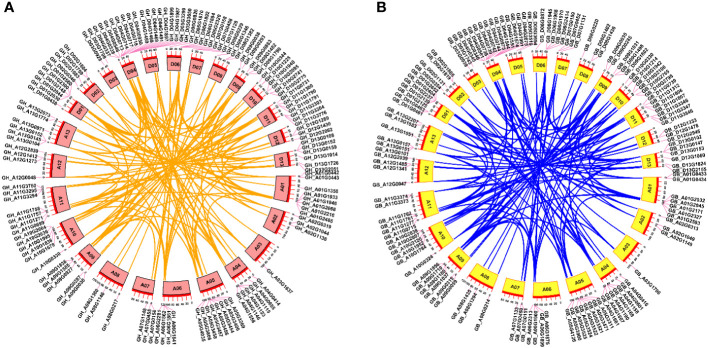
Collinearity analysis of *TBL* gene family from *G. hirsutum* **(A)** and *G. barbadense* **(B)**. Orange and blue lines exhibited the link between paralogous gene pairs.

According to the genome annotation information of *G. hirsutum* and *G. barbadense*, we generated chromosomal distribution maps for the *TBL* gene family (**Figure 2**). The results showed that the 131 *GhTBLs* and 130 *GbTBLs* are unevenly distributed across all 26 chromosomes. For instance, there are 15 *GhTBLs* and 13 *GbTBLs* on chromosome D04, and one *TBL* is found on chromosome A03. The *TBL* family genes have been retained in homologous chromosomes during chromosome doubling events in heterotetraploid cotton and may have evolved to favor the D subgenome. There are certainly other genes that have undergone tandem duplication events, such as GH_A01G0442/GH_A01G0443 and GB_A01G0433 GB_A01G0434 on chromosome A01; GH_A06G1861/GH_A06G1862 and GB_A06G1899/GB_A06G1900 on chromosome A06; and GH_D04G0717/GH_D04G0718 and GB_D04G0345/GB_D04G0346 on chromosome D04. Tandem and segmental duplication are crucial for the generation of new gene family members during the evolutionary process, and the duplicated genes are basically one-to-one correspondence (**Figure 3**; **Supplementary Tables S5****,** **S6**).

### 3.5 *Cis*-acting elements of the *TBL* gene family

*Cis*-elements located in promoters play an important role in the regulation of gene expression. To gain more insight into the functions of *TBL* genes, the *cis*-regulatory elements in the 2000 bp upstream of the transcription start sites of cotton *TBL* genes (**Figure S2**). The study revealed the presence of various response elements, such as light responsive element, defense and stress responsive element involved in drought and low temperature, and hormone responsive element associated with auxin, gibberellin, salicylic acid, abscisic acid and MeJA. It is noteworthy that all *TBL* genes, except GH_D07G0458 and GB_A11G1277, contained multiple light responsive elements. This indicates that the cotton *TBL* genes might play an important role in light-regulated growth and development, and is involved in multiple regulatory pathways when cotton is exposed to environmental stress.

### 3.6 Protein-protein interaction PPI network analysis

The interaction properties of *GhTBL* family proteins in *G. hirsutum* were analyzed using the STRING database. The results are presented in **Supplementary Figure S3A**, which shows 131 amino acid sequences of *TBL* family members in *G. hirsutum* in the protein interaction network, with 24 members, including 5 members with node interactions. Among them, *GhTBL93* (GH_D05G3650) had the most interaction nodes, indicating that the protein requires interaction with other proteins to function properly. The protein nodes and interaction relationships of the *GbTBL* gene family in *G. barbadense* correspond to homologous genes in *G. hirsutum*, which is consistent with those in *G. hirsutum* (**Supplementary Figure S3B**). Among them, *GbTBL92* (GB_D05G3654) has the most interaction nodes, indicating that this protein needs to interact with other proteins to function. There is no significant difference between the protein network of the interaction between *G. hirsutum* and *G. barbadense*.

### 3.7 Association analysis between *TBL* genes and important agronomic traits in cotton

**Figure 4:**
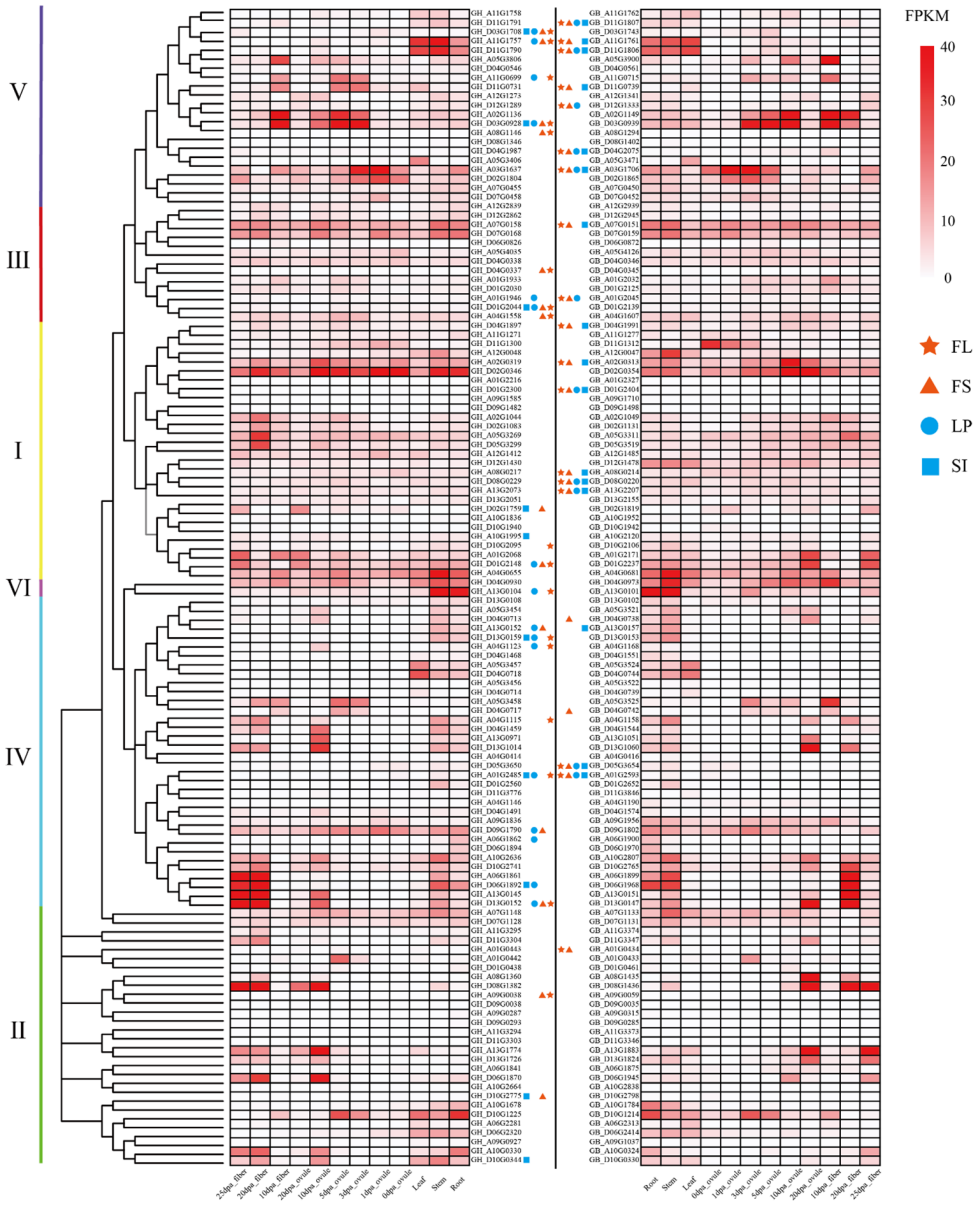
Correlation analysis between *TBL* genes of *G. hirsutum* and *G. barbadense* and fiber length (FL), fiber strength (FS), lint percentage (LP), and seed index (SI). DPA: Days post anthesis. The phylogenetic tree was constructed using the maximum likelihood estimation (ML) method by MEGA 7.0 software. The darker the color, the higher the expression.

To clarify the expression pattern of the cotton *TBL* genes, we analyzed their expression in different tissues: cotton root, stem, leaf, ovule and fiber (**Figure 4**). The *TBL* genes were all expressed in a tissue-specifical manner, indicating their potential involvement in various regulatory pathways during cotton growth and development. In the same group, genes exhibit similar expression patterns, while genes in different groups dis-play distinct expression patterns. During fiber development, *GhTBL* genes in Groups IV and V of *G. hirsutum* are significantly expressed, whereas *GbTBL* genes in Groups II and IV of *G. barbadense* are significantly expressed. In addition, the number of gene expressions in *G. barbadense* is higher than that in *G. hirsutum*. It is worth noting that there is a difference in the peak expression period of homologous genes between *G. hirsutum* and *G. barbadense*. Specifically, *G. barbadense* exhibits a delayed peak expression period during fiber development compared to *G. hirsutum*. To compare the expression trends during fiber development, we selected three pairs of homologous gene pairs in the *G. hirsutum* and *G. barbadense* lineage, the results suggests that *G. barbadense* fibers have a longer elongation and development period, potentially resulting in higher quality fibers (**Supplementary Figure S4**).

By performing association analysis between *TBL* genes and major agronomic traits of *G. hirsutum* and *G. barbadense*, it was found that there is a correlation between *TBL* genes and agronomic traits related to fiber quality and yield (**Supplementary** **Table S7**). As shown in **Figure 4**, there were 17, 14, 16 and 10 genes related to fiber length (FL), fiber strength (FS), lint percentage (LP) and seed index (SI) in *G. hirsutum*, respectively. On the other hand, 18 genes were found to be associated with FL, 20 genes with FS, 11 genes with LP and 16 genes with SI in *G. barbadense*. Regarding the agronomic traits in *G. hirsutum* and *G. barbadense*, 26 and 21 genes respectively were found to be significantly related. The number of genes in *G. hirsutum* was significantly higher than that in *G. barbadense*. Analysis of the homologous genes revealed two pairs of homologous genes associated with FL (GH_A11G1757/GB_A11G1761 and GH_A01G2485/GB_A01G2593) and LP (GH_A01G1946/GB_A01G2045 and GH_A01G2485/GB_A01G2593) traits in both species, and only one pair of homologous genes associated with FS (GH_A11G1757/GB_A11G1761) and SI (GH_A01G2485/GB_A01G2593) traits. Differences in homologous genes between *G. hirsutum* and *G. barbadense*, based on the gene structure of the *TBL* genes, result in differences in trait association. For instance, GH_D11G1790 and GB_D11G1806 exhibit coding sequence differences in Group V (**Supplementary** **Table S7**). They differ in gene length by 123 bp, and GB_D11G1806 is expressed in fibers, while GH_D11G1790 is not. The association between GB_D11G1806 and the four agronomic traits mentioned is significant, whereas GH_D11G1790 shows no significant association with these traits.

### 3.8 Expression profiles of *TBL* gene family under abiotic stresses

**Figure 5:**
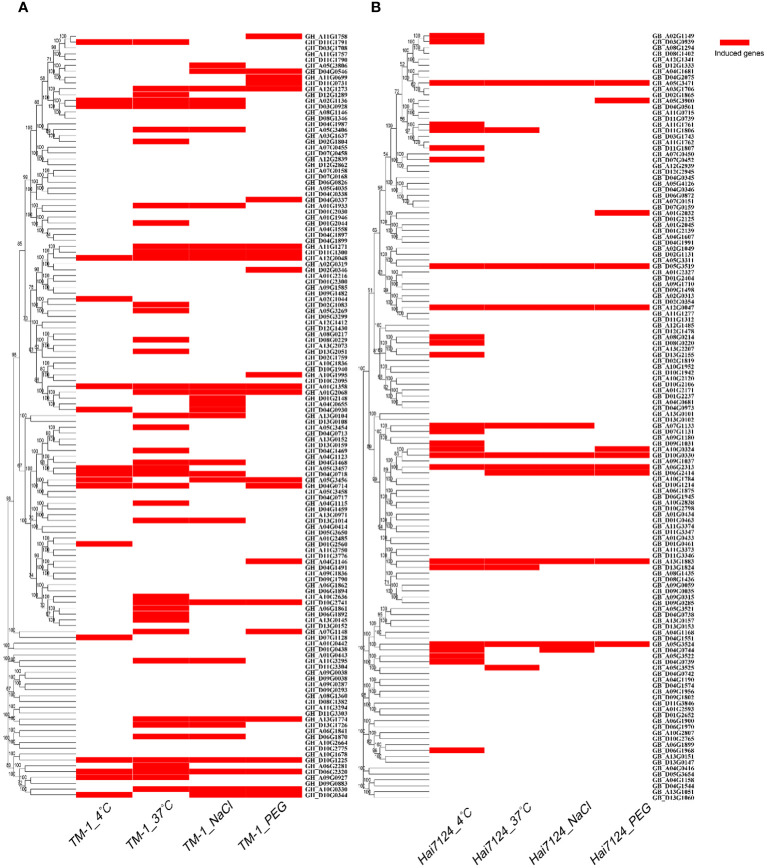
Induced genes of *TBL* gene family from *G hirsutum* **(A)** and *G barbadense* **(B)** under different abiotic stress. After 1 h, 3 h, 6 h, 12 h and 24 h treatment with stress (Low temperature: 4°C; High temperature: 37°C; NaCl: salt stress; PEG: drought stress), up-regulated or down-regulated gene expression was considered to be regulated by abiotic stress.

The RNA-seq data downloaded from the public database was used to further analyze the expression patterns of *TBL* genes were in *G. hirsutum* and *G. barbadense* under different durations of cold, heat, salt, and drought stresses (**Figure 5**). The results showed that most of the *TBL* genes responded to the different stress treatments. In *G. hirsutum*, 59 *TBL* genes were induced by stress under four abiotic stress conditions, while only 29 genes were induced in *G. barbadense*. The induction genes of *G. hirsutum* and *G. barbadense* under four abiotic stress conditions differs among different evolutionary groups, with each having certain preferences. In *G. hirsutum*, the induced genes are mainly distributed in Groups II and IV, while in *G. barbadense*, they are mainly distributed in Groups I, III, and V. The induction of homologous genes in *G. hirsutum* and *G. barbadense* varies under different stress conditions for the same group. For example, *GhTBL119* (GH_D11G1791) is induced under both high and low temperature conditions, whereas the homologous gene *GbTBL118* (GB_D11G1807) is only induced under low temperature conditions. We also found that *GhTBL84* (GH_D04G0930) in group I is induced under both 4°C and salt stress conditions, whereas the homologous gene *GbTBL85* (GB_D04G0973) is not induced under all four abiotic stress conditions. Currently, compared with high temperature, salt and drought stresses, low-temperature stress has a greater impact on cotton during spring emergence, seedling stage and bolling stage, resulting in lower yield and poor quality. Therefore, we verified the role of *GhTBL84* in cotton response to cold stress.

### 3.9 Functional validation of *GhTBL84* response to cold stress

**Figure 6:**
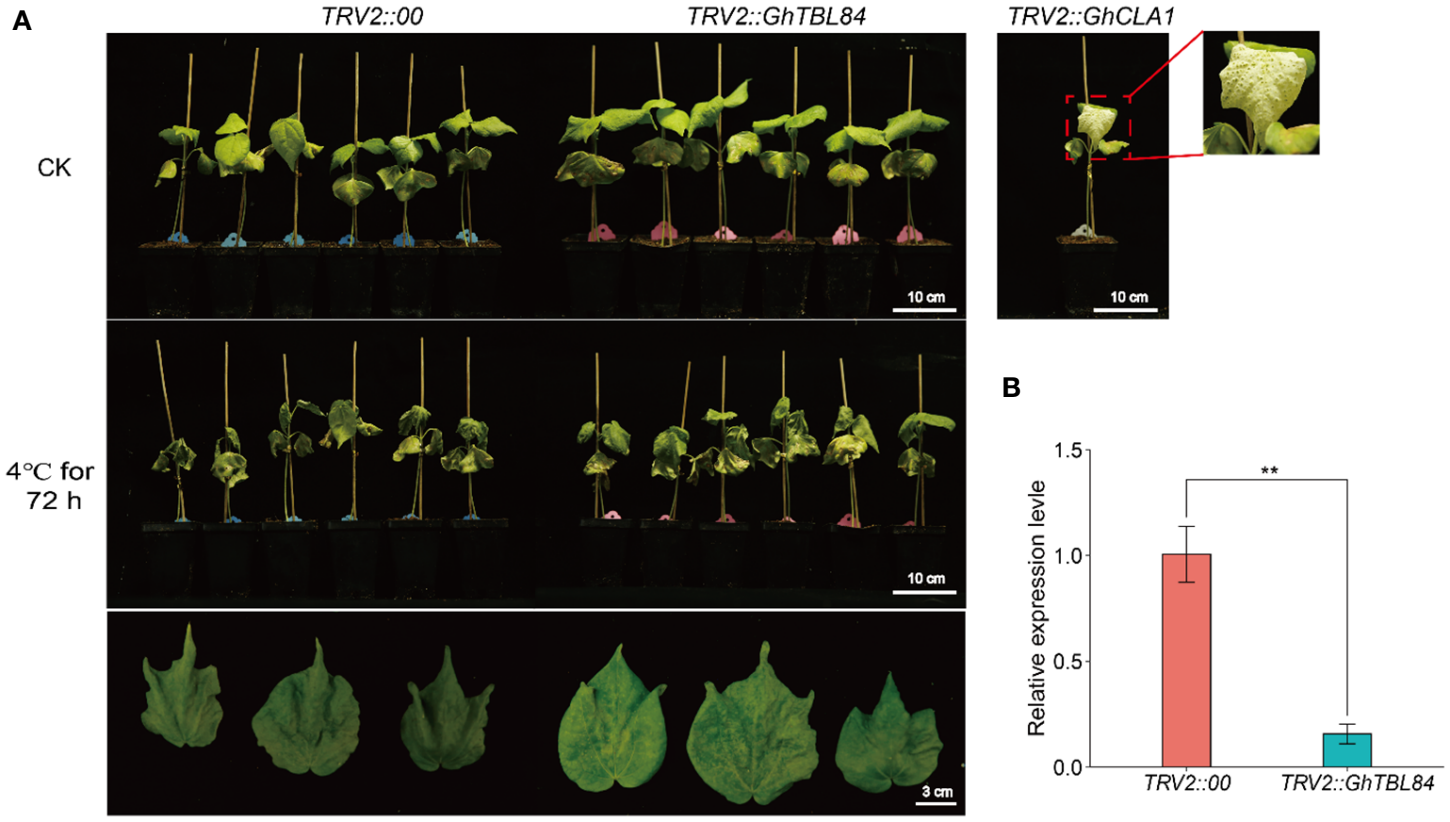
*GhTBL84* responses to cold stress by VIGS validation. **(A)** Representative images showing the phenotypes of *TRV2:00* and *TRV2:GhTBL84* seedlings after cold treatment. **(B)** The relative expression of *GhTBL84* in *TRV2:00* and *TRV2:GhTBL84* seedlings. Values represent means ± SD of three biological replicates. ** indicates significant differences at *p* < 0.01.

Transcriptome data were used to identify the *GhTBL84* gene, which showed a significant change in expression under cold stress (**Supplementary Figure S5**). To investigate the role of *GhTBL84* in the response to cold stress, we utilized the VIGS assay to suppress the expression of *GhTBL84* in cotton TM-1 seedlings. As shown in **Figure 6A**, the *TRV2::GhCLA1* plants exhibited albino phenotypes. The leaf wilting and drooping amplitude of plants inoculated with TRV2::00 was significantly higher than that of *TRV2::GhTBL84* plants under cold treatment. And the expression level of *GhTBL84* in *TRV2::GhTBL84* plants was significantly lower than *TRV2::00* plants (**Figure 6B**), indicating that *GhTBL84* may as a negative regulator response to cold stress.

## 4 Discussion

Cotton is a major cash crop, and its fibers hold an unassailable position in the textile industry. Fibers are formed by the protrusion of a single cell of the ovule epidermis and then differentiates, similar to the formation of epidermal hairs in *A. thaliana*. Therefore, many researchers have used the study of *Arabidopsis* trichomes as a substitute for investigating cotton fibers (Wang et al., 2004; Yang and Ye, 2013). The *TBL* gene families in *Arabidopsis* (Xiong et al., 2013), rice (Gao et al., 2017), quinoa (Ma et al., 2021), and rose (Tian et al., 2021) have been previously identified. However, the function of *TBL* genes in abiotic stress in cotton has not been extensively studied. In this study, we objectively explore the potential function of the cotton *TBL* gene family in cotton fiber development and response to abiotic stress.

### 4.1 Evolutionary analysis of cotton *TBL* gene family

In this study, 131 and 130 *TBL* genes were identified from two tetraploid cotton species, *G. barbadense* (Hai 7124) and *G. hirsutum* (TM-1), respectively. This result is similar to studies reported in cotton, although the number of gene family members is not the same, which may be related to the genomic data and sequence com-parison methods used by the researchers (Kabir et al., 2023; Li Z. et al., 2023). In comparison, there are 45 *TBL* family genes in *A. thaliana* and the number of genes in cotton is 2.8 times higher, suggesting that the *TBL* gene family may have undergone replication events such as whole genome duplication during evolution, resulting in significant gene expansion.

Collinearity analysis showed some occurrence of synonymous mutations. The Ka/Ks values of all gene pairs were less than 1 in *G. hirsutum*. In *G. barbadense*, only one gene pair (GB_D06G1968 and GB_D10G2765) had a Ka/Ks value of 1. 05, while the rest of the genes had a Ka/Ks value of less than 1. This result indicates that this one pair of genes had been under positive selection in the two cotton species and may be the key genes in the evolutionary process, and the rest of the genes are relatively conserved during evolution (Hurst, 2002).

### 4.2 Expression pattern of cotton *TBL* gene family and association analysis of agronomic traits

The developmental stages of cotton fiber include five stages: cell initiation and differentiation, rapid elongation or primary cell wall synthesis, primary to secondary cell wall transition, secondary cell wall synthesis, and fiber maturation (Haigler et al., 2012). Secondary cellulose deposition is a part of the cotton fiber elongation (Hinchliffe et al., 2010).The *TBL* gene is a member of the plant-specific DUF231 structural domain gene family, which is responsible for the initiation of plant trichomes and the O-acetylation pathway in the cell wall. In rice mutant *ostbl1* and *ostbl2*, not only was the plant height significantly reduced, but also the level of cell wall acetylation was affected (Gao et al., 2017). However, the role of *TBLs* in cotton has not been systematically studied.

The *TBL* gene family exhibited tissue-specific expression in cotton, indicating that it may be involved in multiple processes of cotton growth and development. Tissue expression pattern analysis revealed that 87 and 91 *TBL* genes were expressed at different stages of fiber development stage of fiber in *G. barbadense* and *G. hirsutum*, respectively. It suggested that *TBL* family genes may be associated with fiber in tetraploid cultivated cotton species (Kabir et al., 2023; Li J. et al., 2023). The expression peak of *TBL* genes in *G. hirsutum* during fiber development was obviously earlier than that in *G. barbadense*, which indicated that the genes involved in fiber development may be different in two cotton species, and ultimately caused the difference in fiber yield and quality.

Association analysis of *TBL* family genes with important agronomic traits such as FL, FS, SI, and LP in *G. barbadense* and *G. hirsutum*, showed that there were 18 and 20 genes related to yield and quality traits in *G. barbadense*, and 20 and 21 genes related to *G. hirsutum*, respectively. This suggests a higher correlation between the *TBL* family and fiber quality in *G. barbadense*. Previous studies have reported that *GhTBL87* and *GhTBL58* are relevant for fiber length (Kabir et al., 2023), which further confirmed the importance of *TBL* family genes in improving cotton fiber yield and quality. This provided the genetic resources for creating excellent cotton germplasm.

### 4.3 Functional verification of cotton *TBL* gene response to cold stress

Abiotic stresses can also lead to differences in fiber quality. Promoter element perdition showed that *cis*-elements, including light-responsive, hormone-responsive, and low temperature responsive elements, differ in these genes. This suggests *TBL* genes may play a role in response to abiotic stress. For instance, in quinoa, *TBL* is involved in ethylene and salt stress responses (Ma et al., 2021). Similarly, *TBL* genes have been implicated in resistance to phytopathogenic bacteria in *Arabidopsis*, and *RcTBL16* has been shown to be associated with gray mold in rose (Tian et al., 2021). Analysis of the expression patterns of *GhTBL* and *GbTBL* genes under abiotic stress showed that most the genes could be involved in the response to abiotic stress. In addition, more genes were significantly expressed in upland cotton than in island cotton, suggesting their response to abiotic stress may be more pronounced.

Cold stress disrupts normal physiological and metabolic processes in plant, alters cell morphology, and regulates respiration. This results in slow growth, wilting, drooping leaves, and wilting to death, ultimately lead to yield and quality loss (Kidokoro et al., 2022). Seedling emergence and fiber quality in cotton are significantly affected by cold stress. Several studies have been conducted on the physiological and molecular mechanisms of cotton in response to cold stress. For instance, research has shown that exposure to cold temperatures can result in changes to malate metabolism, soluble sugar metabolism, and cellulose synthesis. These changes ultimately lead to a significant reduction in the length of cotton fibers (Wang et al., 2024; Zheng et al., 2012). Similarly, transcriptome sequencing of stress-treated in TM-1 revealed a significant up-regulation of genes related to the ABA signaling pathway under cold stress (Hu et al., 2019). In this study, we found that the *GhTBL84* gene responds to cold stress by characterizing the *TBL* gene family in cotton. Using VIGS technology, it was demonstrated that *GhTBL84* was shown to negatively regulates the cold tolerance in cotton. However, the specific molecular mechanism was not explored in depth. Next, the molecular mechanism of the *GhTBL84* gene involved in cold stress will be further analyzed by yeast two-hybrid (Y2H) and other methods. This will provide excellent genes for the development of cold-tolerant germplasm in cotton.

## Data availability statement

The original contributions presented in the study are included in the article/**Supplementary Material**. Further inquiries can be directed to the corresponding author.

## Author contributions

LF: Writing – original draft, Writing – review & editing, Conceptualization, Data curation, Formal analysis, Funding acquisition, Investigation, Methodology, Project administration, Resources, Software, Supervision, Validation, Visualization. XZ: Data curation, Conceptualization, Writing – review & editing. XM: Data curation, Writing – review & editing. WH: Validation, Writing – review & editing. YX: Formal analysis, Writing – review & editing. SH: Formal Analysis, Writing – review & editing. ZC: Data curation, Writing – review & editing.
