# Supplementary material for: Genome-wide identification of TBL gene family and functional analysis of GhTBL84 under cold stress in cotton
Source: Front Plant Sci. 2024 Jun 18;15:1431835. doi: 10.3389/fpls.2024.1431835 (PMC11217346; doi:10.3389/fpls.2024.1431835)
Supplement: Supplementary file 1 [file DataSheet_1.pdf]

Supplementary Material

Genome-wide identification of *TBL* gene family and functional analysis of *GhTBL84* under cold stress in cotton

Xiaoqing Zhu[1], Xiaowei Ma[1], Wanying Hu[1], Yulin Xing[1], Shengcai Huang[1], Zequan Chen[1,2] and Lei Fang[1,2,*]

[1]Zhejiang Provincial Key Laboratory of Crop Genetic Resources, Institute of Crop Science, Plant Precision Breeding Academy, College of Agriculture and Biotechnology, Zhejiang University, Hangzhou 310058, China
[2]Hainan Institute of Zhejiang University, Sanya 572025, China

[*] Correspondence:
Lei Fang
fangl@zju.edu.cn

Supplemental Figures

Figure S1. Phylogenetic trees of *TBL* gene family, protein motif and gene structure from *G. hirsutum* (Figure S1A, B) and *G. barbadense* (Figure S1C, D).

Figure S2. *Cis*-acting elements of the promoter of *TBL* gene family from *G. hirsutum* (A) and *G. barbadense* (B).

Figure S3. Analysis of TBL family protein interaction network in *G. hirsutum* (A) and *G. barbadense* (B).

Figure S4. Comparison of expression trends of selected three pairs of homologous genes in *G. hirsutum* and *G. barbadense* during fiber development. (A)GH_A10G2068/GB_A01G2171; (B) GH_A13G1774/GB_A13G1883; (C)GH_D01G2148/GB_D01G2237.

Figure S5. The expression patterns of *GhTBL84* under cold stress. Asterisks indicate statistical significance (*, $P < 0.05$; **, $P < 0.01$; t-test).

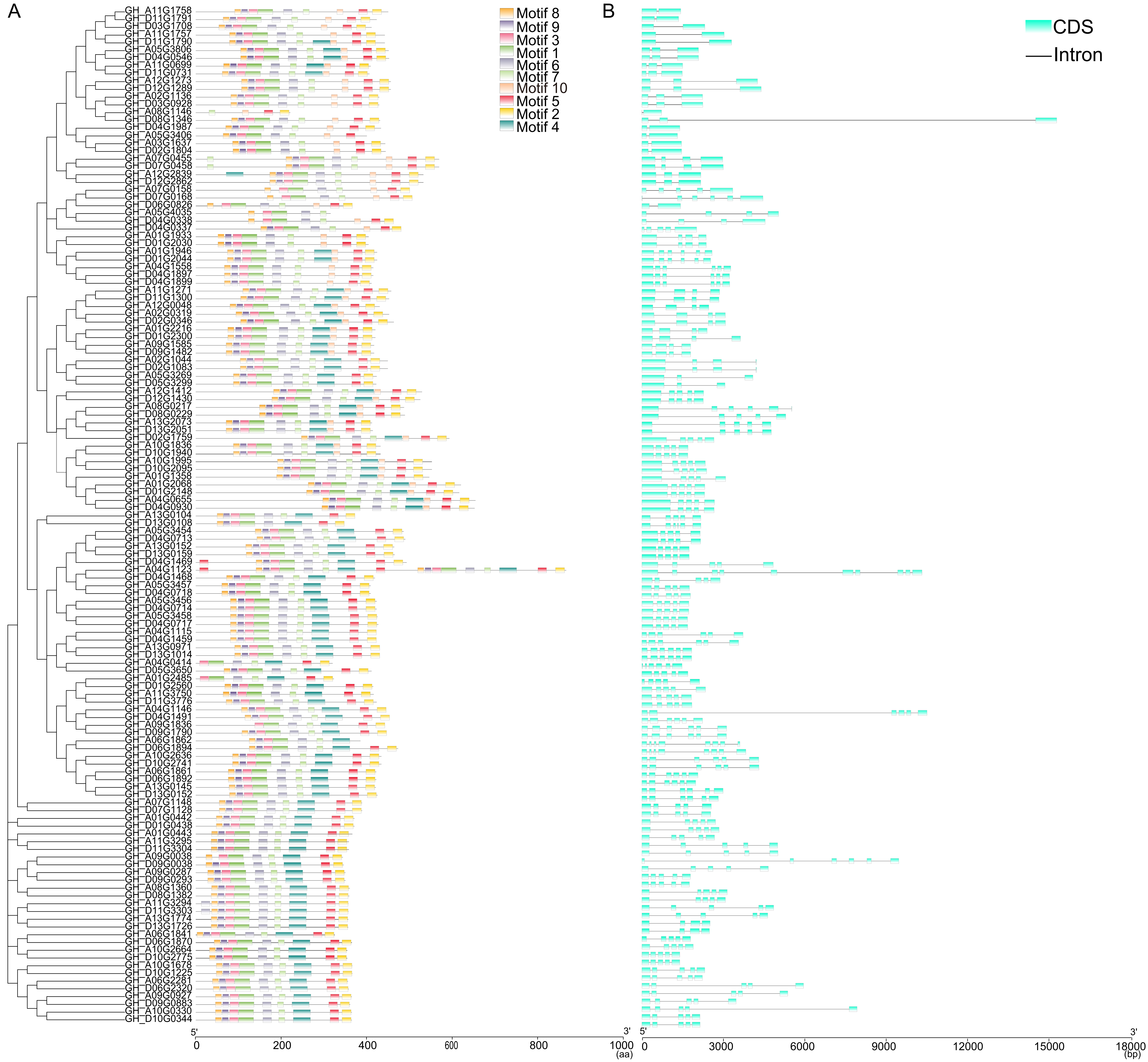
A
B
Motif 8
Motif 9
Motif 3
Motif 1
Motif 6
Motif 7
Motif 10
Motif 5
Motif 2
Motif 4
CDS
Intron
GH_A11G1758
GH_D11G1791
GH_D03G1708
GH_A11G1757
GH_D11G1790
GH_A05G3806
GH_D04G0546
GH_A11G0699
GH_D11G0731
GH_A12G1273
GH_D12G1289
GH_A02G1136
GH_D03G0928
GH_A08G1146
GH_D08G1346
GH_D04G1987
GH_A05G3406
GH_A03G1637
GH_D02G1804
GH_A07G0455
GH_D07G0458
GH_A12G2839
GH_D12G2862
GH_A07G0158
GH_D07G0168
GH_D06G0826
GH_A05G4035
GH_D04G0338
GH_D04G0337
GH_A01G1933
GH_D01G2030
GH_A01G1946
GH_D01G2044
GH_A04G1558
GH_D04G1897
GH_D04G1899
GH_A11G1271
GH_D11G1300
GH_A12G0048
GH_A02G0319
GH_D02G0346
GH_A01G2216
GH_D01G2300
GH_A09G1585
GH_D09G1482
GH_A02G1044
GH_D02G1083
GH_A05G3269
GH_D05G3299
GH_A12G1412
GH_D12G1430
GH_A08G0217
GH_D08G0229
GH_A13G2073
GH_D13G2051
GH_D02G1759
GH_A10G1836
GH_D10G1940
GH_A10G1995
GH_D10G2095
GH_A01G1358
GH_A01G2068
GH_D01G2148
GH_A04G0655
GH_D04G0930
GH_A13G0104
GH_D13G0108
GH_A05G3454
GH_D04G0713
GH_A13G0152
GH_D13G0159
GH_D04G1469
GH_A04G1123
GH_D04G1468
GH_A05G3457
GH_D04G0718
GH_A05G3456
GH_D04G0714
GH_A05G3458
GH_D04G0717
GH_A04G1115
GH_D04G1459
GH_A13G0971
GH_D13G1014
GH_A04G0414
GH_D05G3650
GH_A01G2485
GH_D01G2560
GH_A11G3750
GH_D11G3776
GH_A04G1146
GH_D04G1491
GH_A09G1836
GH_D09G1790
GH_A06G1862
GH_D06G1894
GH_A10G2636
GH_D10G2741
GH_A06G1861
GH_D06G1892
GH_A13G0145
GH_D13G0152
GH_A07G1148
GH_D07G1128
GH_A01G0442
GH_D01G0438
GH_A01G0443
GH_A11G3295
GH_D11G3304
GH_A09G0038
GH_D09G0038
GH_A09G0287
GH_D09G0293
GH_A08G1360
GH_D08G1382
GH_A11G3294
GH_D11G3303
GH_A13G1774
GH_D13G1726
GH_A06G1841
GH_D06G1870
GH_A10G2664
GH_D10G2775
GH_A10G1678
GH_D10G1225
GH_A06G2281
GH_D06G2320
GH_A09G0927
GH_D09G0883
GH_A10G0330
GH_D10G0344
5'
3'
0
200
400
600
800
1000
(aa)
5'
3'
0
3000
6000
9000
12000
15000
18000
(bp)

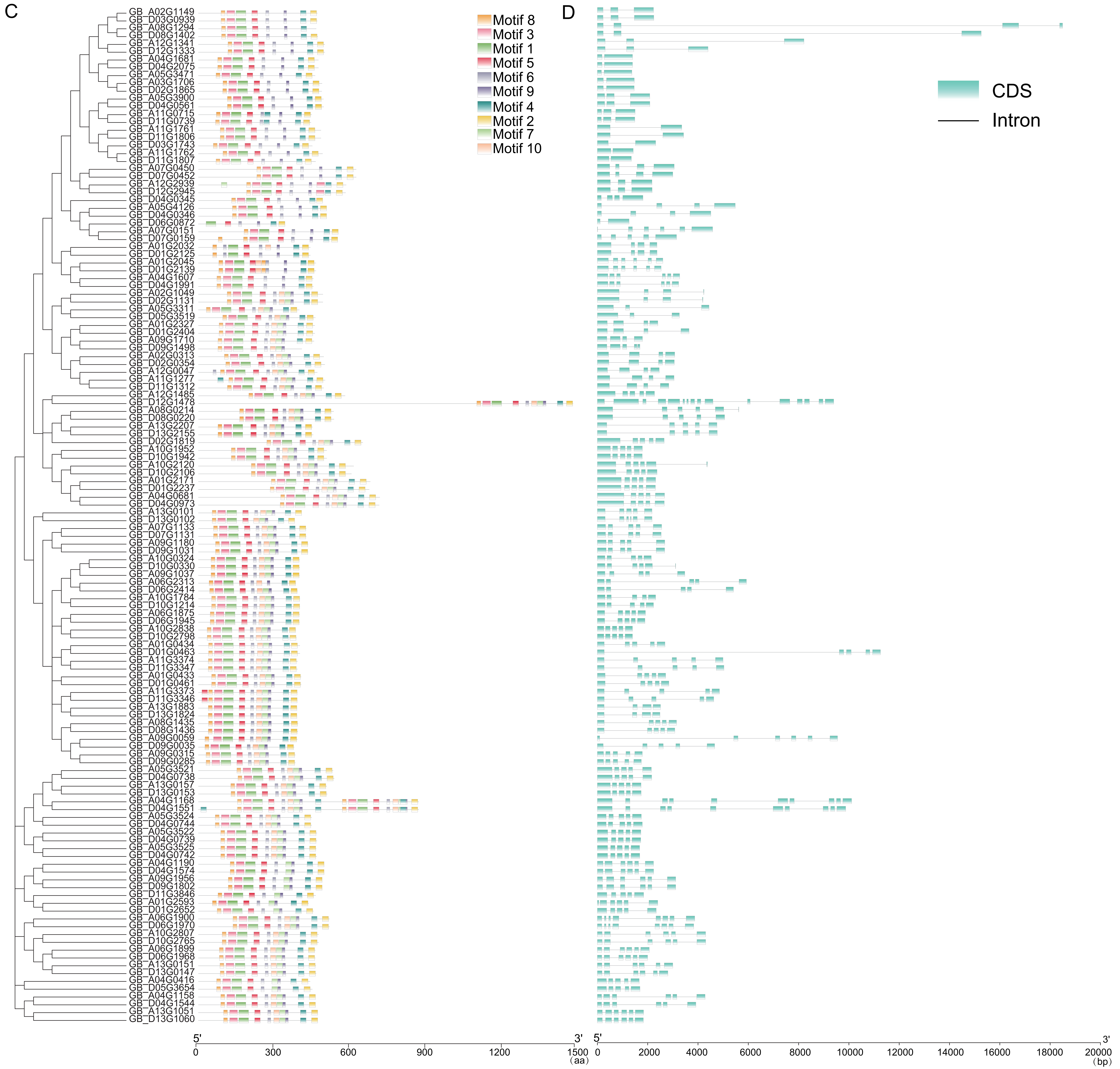

Figure S1. Phylogenetic trees of *TBL* gene family, protein motif and gene structure from *G. hirsutum* (A, B) and *G. barbadense* (C, D).

A

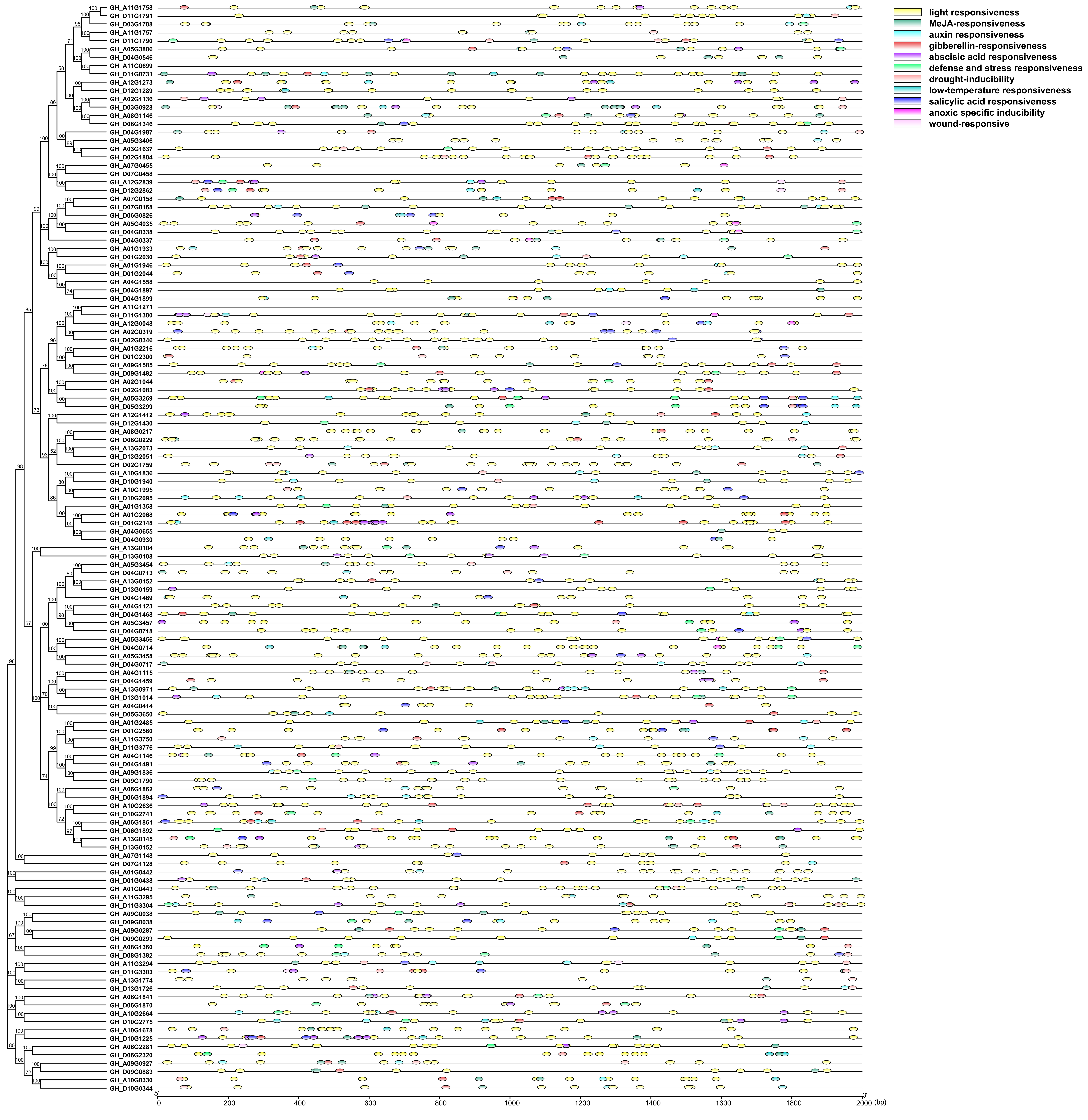

light responsiveness
MeJA-responsiveness
auxin responsiveness
gibberellin-responsiveness
abscisic acid responsiveness
defense and stress responsiveness
drought-inducibility
low-temperature responsiveness
salicylic acid responsiveness
anoxic specific inducibility
wound-responsive
GH_A11G1758
GH_D11G1791
GH_D03G1708
GH_A11G1757
GH_D11G1790
GH_A05G3806
GH_D04G0546
GH_A11G0699
GH_D11G0731
GH_A12G1273
GH_D12G1289
GH_A02G1136
GH_D03G0928
GH_A08G1146
GH_D08G1346
GH_D04G1987
GH_A05G3406
GH_A03G1637
GH_D02G1804
GH_A07G0455
GH_D07G0458
GH_A12G2839
GH_D12G2862
GH_A07G0158
GH_D07G0168
GH_D06G0826
GH_A05G4035
GH_D04G0338
GH_D04G0337
GH_A01G1933
GH_D01G2030
GH_A01G1946
GH_D01G2044
GH_A04G1558
GH_D04G1897
GH_D04G1899
GH_A11G1271
GH_D11G1300
GH_A12G0048
GH_A02G0319
GH_D02G0346
GH_A01G2216
GH_D01G2300
GH_A09G1585
GH_D09G1482
GH_A02G1044
GH_D02G1083
GH_A05G3269
GH_D05G3299
GH_A12G1412
GH_D12G1430
GH_A08G0217
GH_D08G0229
GH_A13G2073
GH_D13G2051
GH_D02G1759
GH_A10G1836
GH_D10G1940
GH_A10G1995
GH_D10G2095
GH_A01G1358
GH_A01G2068
GH_D01G2148
GH_A04G0655
GH_D04G0930
GH_A13G0104
GH_D13G0108
GH_A05G3454
GH_D04G0713
GH_A13G0152
GH_D13G0159
GH_D04G1469
GH_A04G1123
GH_D04G1468
GH_A05G3457
GH_D04G0718
GH_A05G3456
GH_D04G0714
GH_A05G3458
GH_D04G0717
GH_A04G1115
GH_D04G1459
GH_A13G0971
GH_D13G1014
GH_A04G0414
GH_D05G3650
GH_A01G2485
GH_D01G2560
GH_A11G3750
GH_D11G3776
GH_A04G1146
GH_D04G1491
GH_A09G1836
GH_D09G1790
GH_A06G1862
GH_D06G1894
GH_A10G2636
GH_D10G2741
GH_A06G1861
GH_D06G1892
GH_A13G0145
GH_D13G0152
GH_A07G1148
GH_D07G1128
GH_A01G0442
GH_D01G0438
GH_A01G0443
GH_A11G3295
GH_D11G3304
GH_A09G0038
GH_D09G0038
GH_A09G0287
GH_D09G0293
GH_A08G1360
GH_D08G1382
GH_A11G3294
GH_D11G3303
GH_A13G1774
GH_D13G1726
GH_A06G1841
GH_D06G1870
GH_A10G2664
GH_D10G2775
GH_A10G1678
GH_D10G1225
GH_A06G2281
GH_D06G2320
GH_A09G0927
GH_D09G0883
GH_A10G0330
GH_D10G0344
5'
3'
0
200
400
600
800
1000
1200
1400
1600
1800
2000 (bp)

B

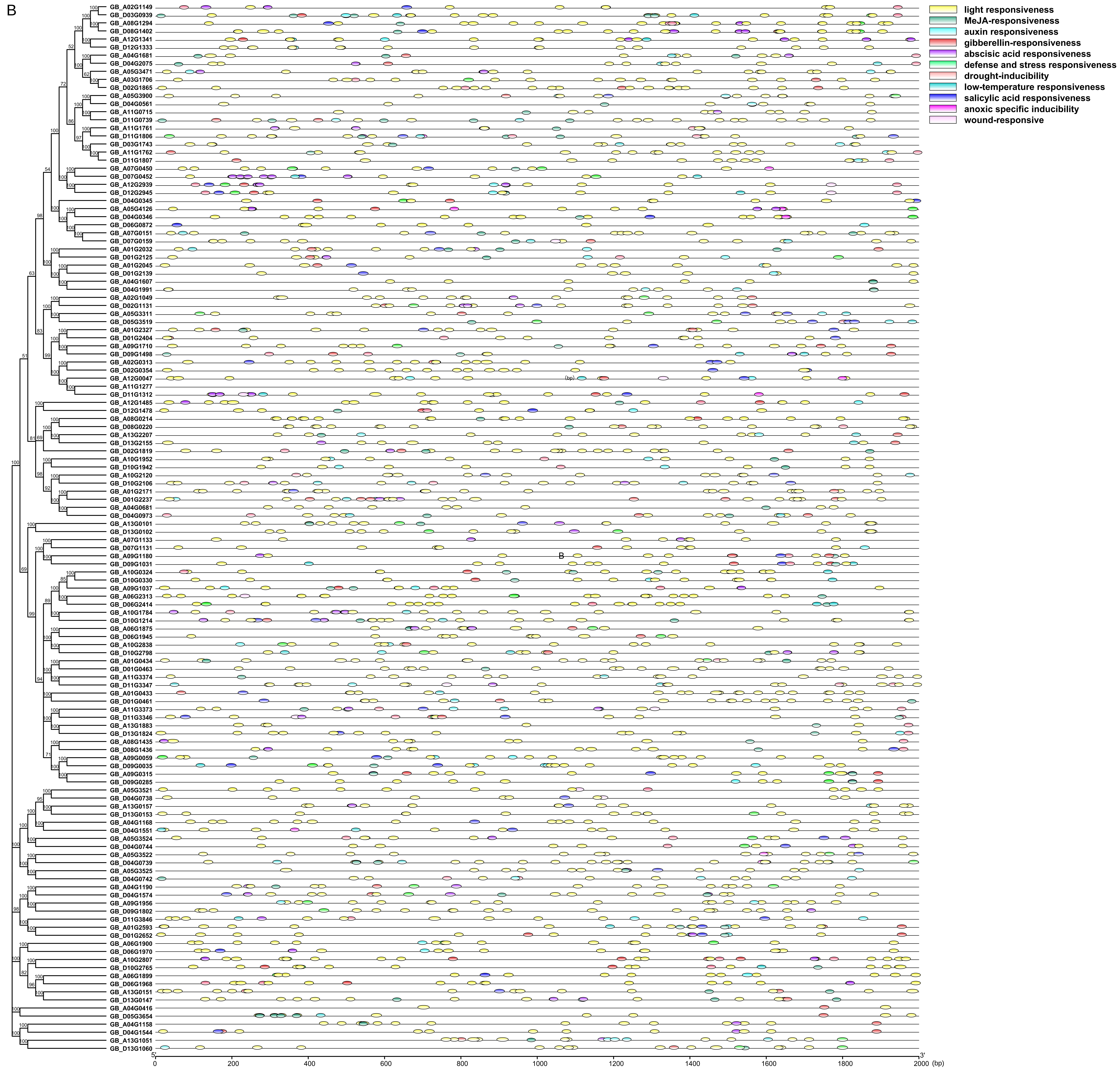

Figure S2: *Cis*-acting elements of the promoter of *TBL* gene family from *G. hirsutum* (A) and *G. barbadense* (B).

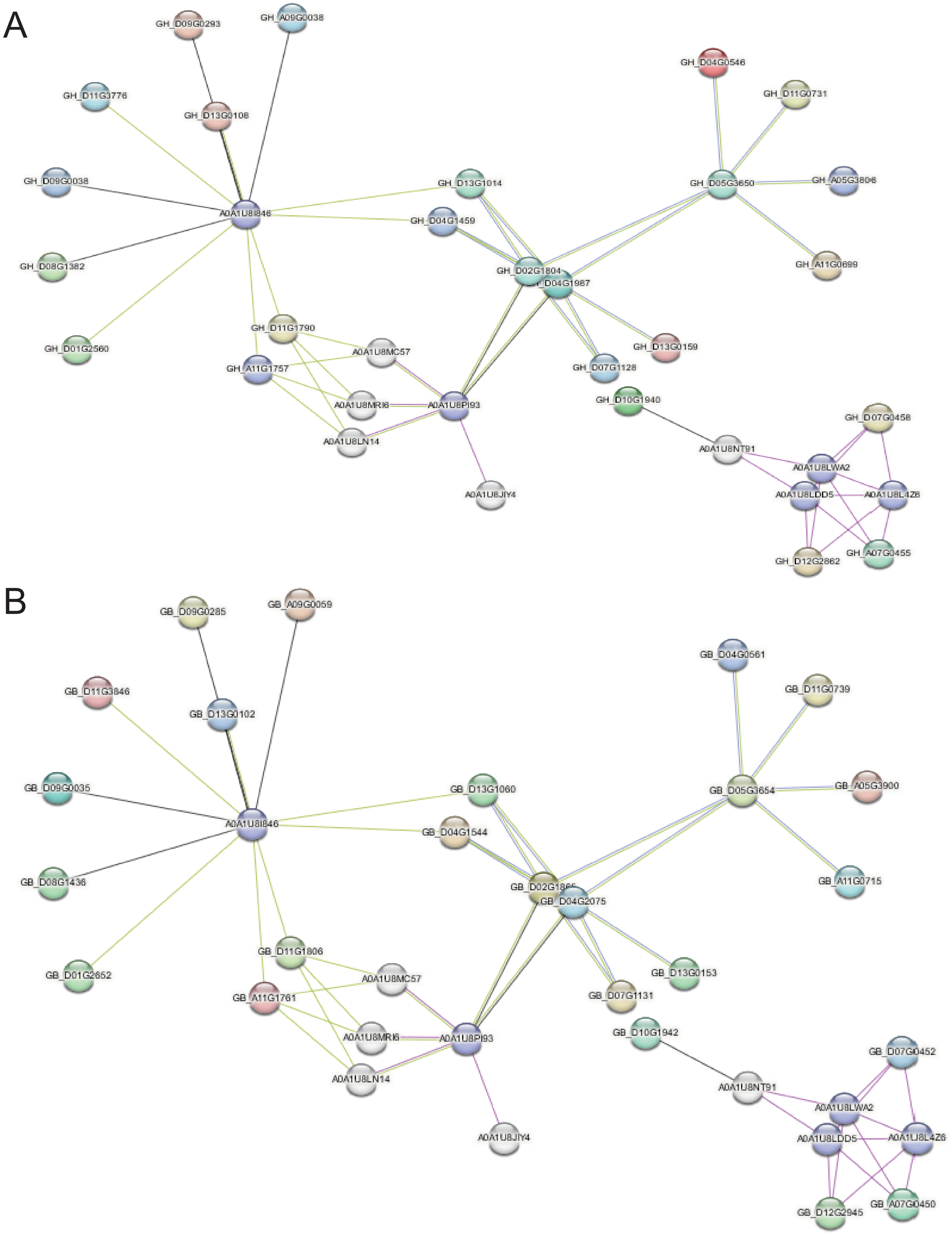

Figure S3. Analysis of TBL family protein interaction network in *G. hirsutum* (A) and *G. barbadense* (B).

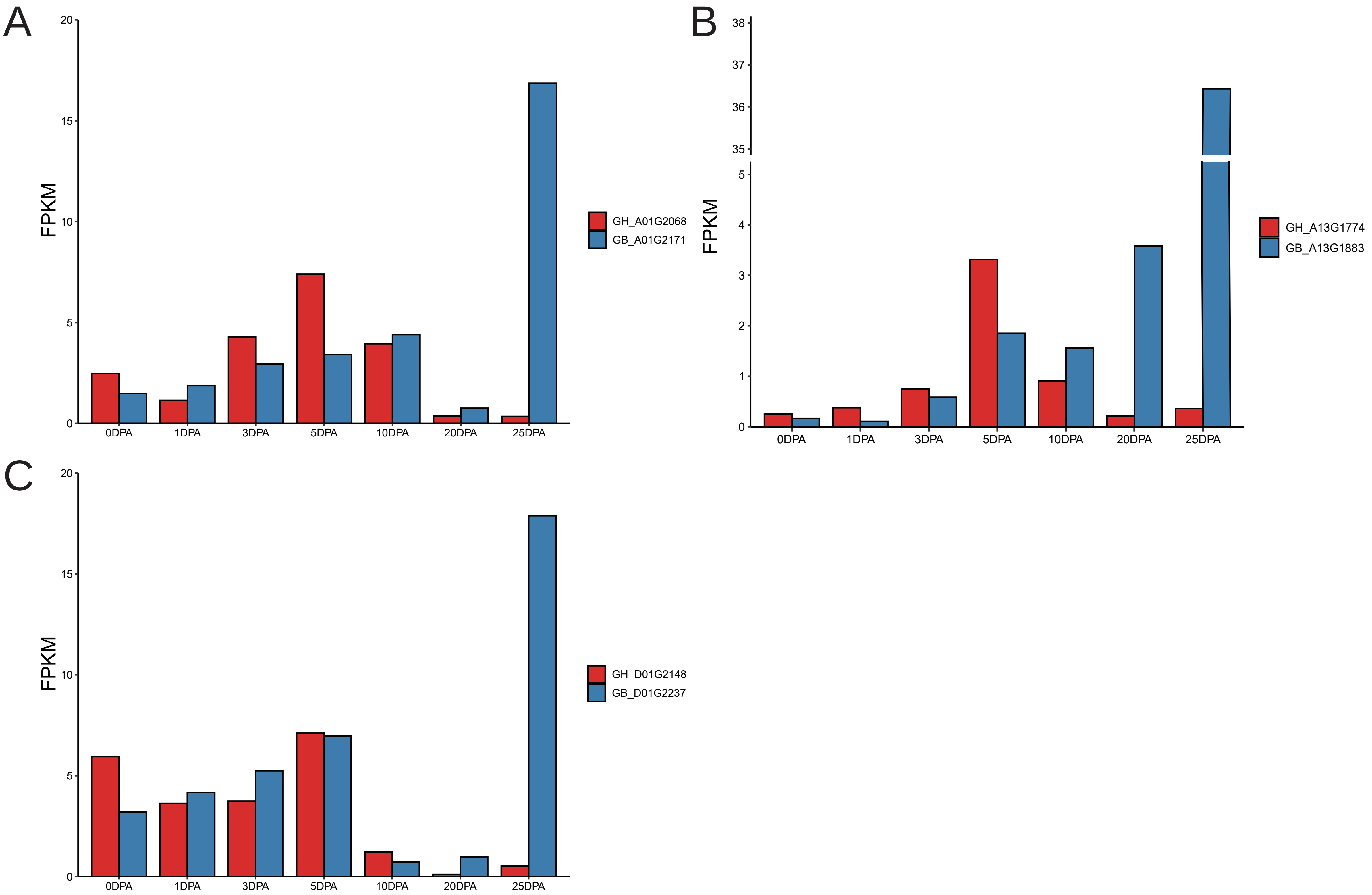

Figure S4. Comparison of expression trends of selected three pairs of homologous genes in *G. hirsutum* and *G.barbadense* during fiber development.(A)GH_A10G2068/GB_A01G2171; (B)GH_A13G1774/GB_A13G1883; (C)GH_D01G2148/GB_D01G2237.

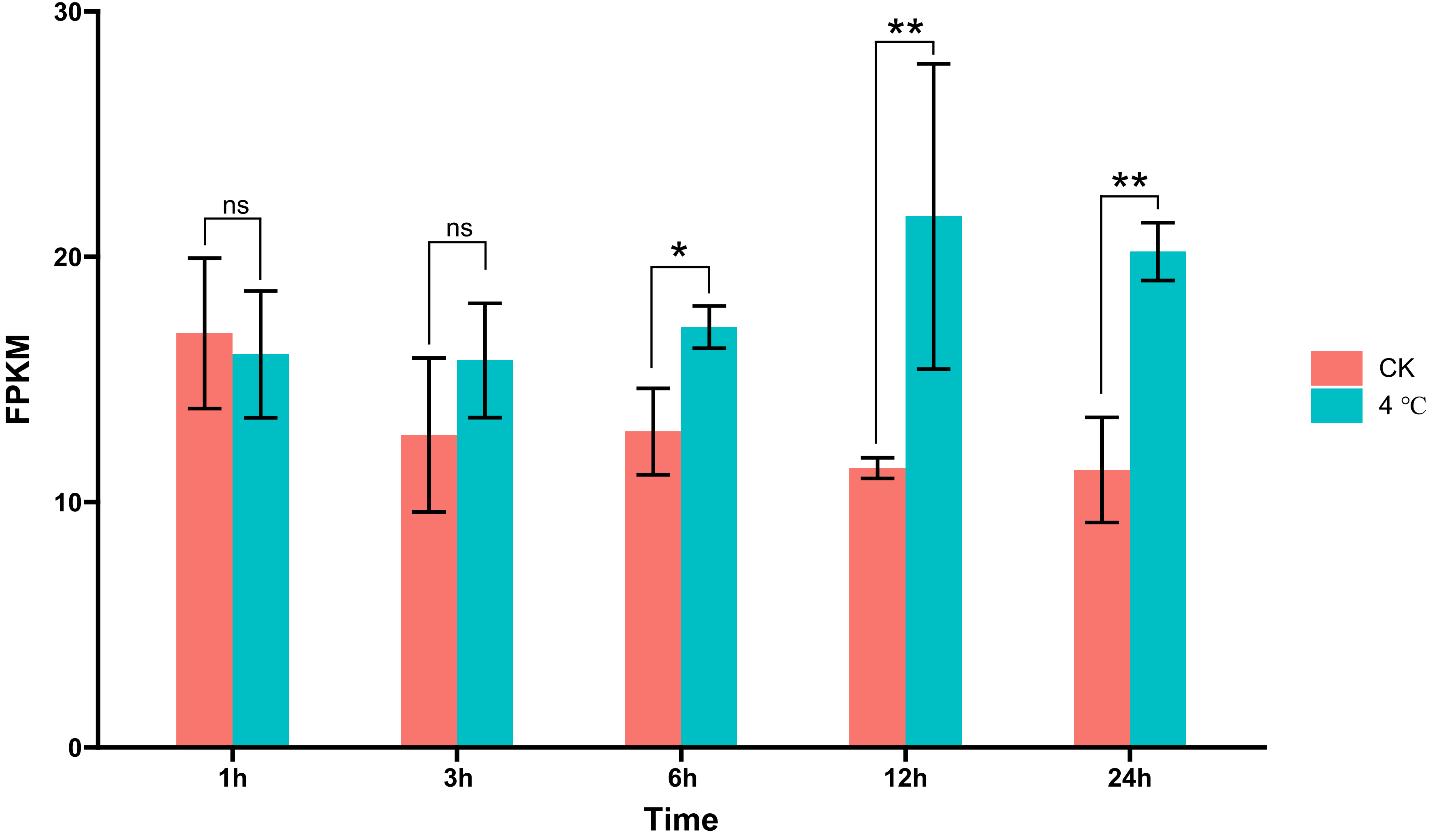

Figure S5. The expression patterns of *GhTBL84* under cold stress. Asterisksindicate statistical significance (*, *P* < 0.05; **, *P* < 0.01; t-test).
